# Eukaryotic Box C/D methylation machinery has two non-symmetric protein assembly sites

**DOI:** 10.1038/s41598-021-97030-y

**Published:** 2021-09-02

**Authors:** Simone Höfler, Peer Lukat, Wulf Blankenfeldt, Teresa Carlomagno

**Affiliations:** 1 Institute for Organic Chemistry and Centre of Biomolecular Drug Research (BMWZ), Leibniz University Hannover, 30167 Hannover, Lower Saxony Germany; 2 Department of Structure and Function of Proteins, Helmholtz Centre of Infection Research, 38124 Braunschweig, Lower Saxony Germany; 3 Group of NMR-Based Structural Chemistry, Helmholtz Centre of Infection Research, 38124 Braunschweig, Lower Saxony Germany; 4 Institute for Biochemistry, Biotechnology and Bioinformatics, Technische Universität Braunschweig, Spielmannstr. 7, 38106 Braunschweig, Germany

**Keywords:** Biochemistry, Molecular biology, Structural biology

## Abstract

Box C/D ribonucleoprotein complexes are RNA-guided methyltransferases that methylate the ribose 2’-OH of RNA. The central ‘guide RNA’ has box C and D motifs at its ends, which are crucial for activity. Archaeal guide RNAs have a second box C’/D’ motif pair that is also essential for function. This second motif is poorly conserved in eukaryotes and its function is uncertain. Conflicting literature data report that eukaryotic box C’/D’ motifs do or do not bind proteins specialized to recognize box C/D-motifs and are or are not important for function. Despite this uncertainty, the architecture of eukaryotic 2’-*O*-methylation enzymes is thought to be similar to that of their archaeal counterpart. Here, we use biochemistry, X-ray crystallography and mutant analysis to demonstrate the absence of functional box C’/D’ motifs in more than 80% of yeast guide RNAs. We conclude that eukaryotic Box C/D RNPs have two non-symmetric protein assembly sites and that their three-dimensional architecture differs from that of archaeal 2’-*O*-methylation enzymes.

## Introduction

Chemical modifications of RNA base or ribose diversify the structures and functions of RNA^1–3^. Ribosomal RNA (rRNA) is methylated at the 2’-OH position (2’-*O*-methylation) in all kingdoms of life^4–7^; this modification ensures correct rRNA folding, structural and thermodynamic stability, translational efficiency and proof-reading^8–11^. In humans, deregulation of 2’-*O*-methylation is involved in several diseases, including cancer and neuropathologies^12,13^.

The positions of the 2’-*O*-Me modifications are well defined in rRNAs and those located in functional regions of the ribosome are highly conserved from bacteria to eukaryotes^14–17^. Ribonucleoprotein (RNP) complexes called Box C/D RNPs methylate 2’-OH groups during ribosome biogenesis^18–21^. These RNPs assemble around a small nucleolar (sno) ‘guide’ RNA that recognizes individual substrates by base complementarity.

**Figure 1:**
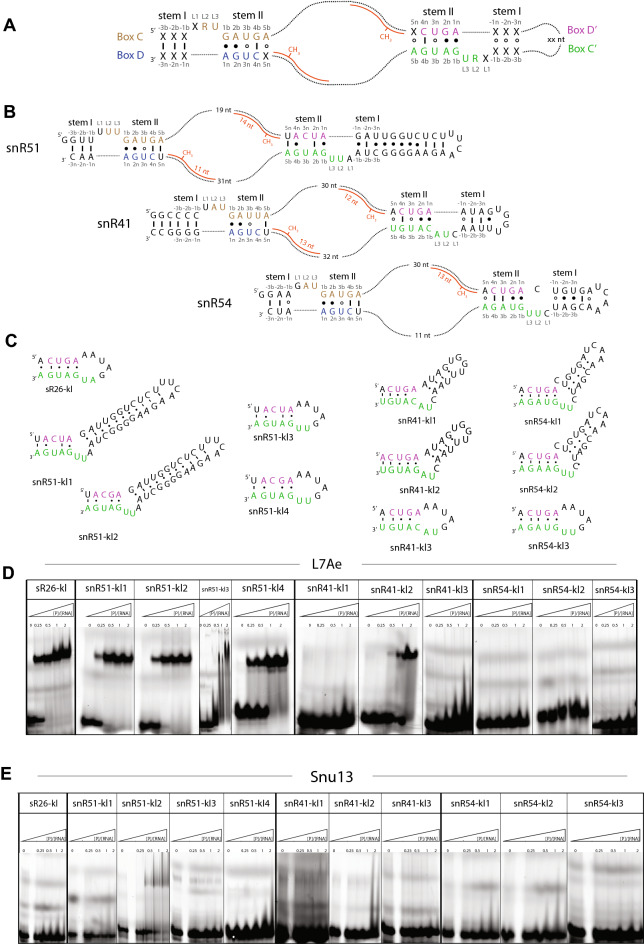
Snu13 binds none of the non-canonical box C’/D’ motifs. (**A**) Schematic representation of box C/D guide snoRNA. The consensus sequences of the conserved motifs box C and D are shown in brown and blue, respectively; the consensus sequences of the poorly conserved motifs box C’ and D’ are shown in green and purple, respectively. The secondary structure elements flanking the box C/D (or box C’/D’) motifs are named stem I and stem II. The numbering of the nucleotides in and around the consensus sequences is given above each nucleotide. In kink-loop (k-loop) RNAs, the stem I flanking the box C’/D’ motif is substituted by a loop. The guide sequences between box C and box D’ and between box C’ and D recognize complementary substrate rRNA sequences (red). (**B**) Predicted secondary structure of yeast box C/D guide snoRNAs snR51, snR41 and snR54; the box C/D and box C’/D’ motifs are colored as in panel A. The guide sequences are shown base-paired with the rRNA substrates (red); the length of each complementary guide sequence is given below the substrate RNAs. (**C**) Predicted secondary structures of the RNAs designed to represent archaeal and eukaryotic guide RNAs at the box C’/D’ site. K-loop sR26-kl1 is derived from archaeal *Pf* guide RNA sR26. Non-canonical k-turns snR51-kl1, snR41-kl1 and snR54-kl1 are derived from eukaryotic *Sc* guide RNA snR51, snR41 and snR54, respectively; snR51kl-2, snR51-kl3 and snR51-kl4 are mutants of snR51-kl1; snR41-kl2 and sn41-kl3 are mutants of snR41-kl1; snR54-kl2 and snR54-kl3 are mutants of snR54-kl1. Box C’ and D’ are colored in green and purple, respectively. (**D**) Electrophoretic mobility shift assays (EMSAs) of the 5’-Cy5 fluorescently labeled RNAs of panel C in the presence of archaeal L7Ae. (E) EMSAs of the 5’-Cy5 fluorescently labeled RNAs of panel C in the presence of yeast Snu13. In (**D**, **E**) the first lane of each panel contains 10 pmol of RNA only. The ratio of total protein and total RNA concentrations ([P]/[RNA]) is given above each lane. The RNA concentration in each of the five lanes is in the order: 2, 1.9, 1.8, 1.7 and 1.4 µM. The figure was prepared with Adobe Illustrator (Adobe Inc., 2020/21; https://adobe.com/products/illustrator).

Guide RNAs form a kink-turn (k-turn) three-dimensional structure that binds the Snu13 (or 15.5 kDa, now named SNU13) protein^22^. The k-turn structure takes its name from the characteristic 50° kink of the RNA backbone (Supplementary Fig. 1); it is formed by a box C sequence motif (5’-RUGAUGA) at the 5’ end of the guide RNA and a box D sequence motif (5’-CUGA) at the 3’ end (Fig. 1A)^19,20^. Two sheared G•A base-pairs flanking the kink in stem II are typical of these structures^23,24^. Archaeal guide RNAs have similar additional motifs in the interior of the sequence, called boxes C’ and D’ (Fig. 1A)^25,26^; the motifs fold in either an internal k-turn or in a k-loop structure, depending on whether the kink is flanked by an RNA helix (stem I) or a loop^27^. These structures provide a second binding site for L7Ae, the archaeal orthologue of Snu13. In eukaryotes, however, the predicted box C’/D’ motifs diverge from the consensus sequence and often lack one of the two sheared G•A base-pairs. They are thought to fold in a k-turn structure, as in archaea; however, the evidence for this is not conclusive and their ability to recruit a second copy of Snu13 to snoRNA is controversial^28,29^.

The methyltransferase Nop1 (or fibrillarin) is brought to the Snu13–snoRNA complex by the two scaffolding proteins Nop56 and Nop58, which form a heterodimer through their coiled-coil domains. In cross-linking studies^28^, the C-terminal domain of Nop58 was found to bind to the composite surface formed by the RNA box C/D motif and Snu13 (Supplementary Fig. 2), whereas the C-terminal domain of Nop56 was found near the predicted box C’/D’ motif. The N-terminal domains of the eukaryotic Nop56–Nop58 heterodimer bind the methyltransferase Nop1 in yeast (fibrillarin in humans) and recruit it to the RNP (Supplementary Fig. 2).

Unfortunately, all attempts to reconstitute a methylation-competent and homogeneous eukaryotic Box C/D RNP in vitro have failed, precluding structural studies. Instead, there are numerous structures of archaeal Box C/D RNPs^30–34^. In archaea, the homodimer Nop5_2_ substitutes Nop56–Nop58; each Nop5 monomer recognizes one k-turn or k-loop L7Ae–RNA site, with the coiled-coil dimerization domains running roughly along the axis of the guide RNA (Supplementary Fig. 2). The two fibrillarin copies, recruited by the Nop5 N-terminal domains, transfer a methyl group to the ribose 2’-OH of two substrate RNAs, each base-paired with one of the two guide-RNA sequences upstream of box D and of box D’. The methyl group transfer is selective and occurs to the fifth nucleotide upstream of both box D and box D’. The substrate-loaded archaeal Box C/D RNP may adopt a monomeric or dimeric form, depending on the guide RNA sequence (Supplementary Fig. 2)^35^. In both cases, the restraint imposed on the RNA geometry by the length of the coiled-coil domains, which run between the L7Ae-bound box C/D and box C’/D’ motifs, specifies the methylation position on the substrate RNAs.

Because of the lack of a three-dimensional structure of a eukaryotic, methylation-competent Box C/D enzyme, the archaeal monomeric Box C/D RNP is generally used as a proxy of the eukaryotic enzyme. The assumption that eukaryotic and archaeal methylation machineries have similar architectures is reinforced by the electron microscopy structure of the eukaryotic U3 snoRNP bound to the pre-ribosomal 90S subunit^36^. In this structure, the U3 snoRNP is symmetric and features two identical protein assemblies at the box C’/D (corresponding to box C/D) and box B/C (with the sequence of a canonical box C’/D’) sites. However, the U3 snoRNP does not function as a methyltransferase, but acts as a chaperone to aid rRNA folding^37,38^.

In archaea, the C-terminal domain of Nop5 is incapable of binding box C/D motifs in the absence of the primary binding protein L7Ae. Because of the high sequence similarity between archaeal and eukaryotic Nop5 and Nop56/58 C-terminal domains (~ 60% similarity and 42% identity between *Pyrococcus furiosus* Nop5 and yeast Nop56/Nop58 C-terminal domains), and because the interaction surfaces between the Nop56/Nop58 C-terminal domains and both the RNA kink-turn and Snu13 in the U3 snoRNP structure are similar to those of archaeal Box C/D complexes, eukaryotic Nop56/Nop58 C-terminal domains should also be unable to bind box C/D motifs in the absence of Snu13^39^. Thus, if eukaryotic box C’/D’ motifs do not bind Snu13, it remains unclear how the C-terminal domain of Nop56 recognizes the RNA at this site and whether the eukaryotic methylation machinery adopts the symmetric architecture seen in archaeal Box C/D complexes and in the eukaryotic U3 snoRNP.

To answer the question whether eukaryotic box C’/D’ motifs are able to recruit Snu13 either in isolation or in the context of the fully assembled Box C/D complex, we study three yeast snoRNAs with putative box C’/D’ motifs predicted by bioinformatics analysis. We define a functional box C’/D’ motif as two juxtaposing sequences capable of base-pairing as in Fig. 1A and of folding in the kinked three-dimensional structure typical of these sequence motifs. To verify whether the predicted sequences are functional box C’/D’ motifs, we test their ability to bind the archaeal protein L7Ae, which is a strong binder and inducer of k-turn-like structures^40^. We find that the motifs are either unable to bind L7Ae or bind with a different structure from that predicted; none of them recognizes the eukaryotic protein Snu13. With a systematic mutational analysis, we identify the sequence elements that are indispensable to yield a functional box C’/D’ motif and we predict that more than 80% of the snoRNA sequences annotated as “non-canonical” box C’/D’ adopt neither the secondary nor the three-dimensional structure typical of this motif. It follows that most of the predicted box C’/D’ motifs of eukaryotic snoRNAs do not bind Snu13 and thus are not functional. Hence, the eukaryotic 2’-*O*-methylation machinery is asymmetric and the recruitment of Nop56 to this site must occur differently from the recruitment of Nop58 to the box C/D site. Our work challenges the common assumption that the substrate-loaded, monomeric RNP of archaea is a good proxy for the eukaryotic RNP and points to divergent mechanisms of activity and regulation of methylation of the site upstream of box D’ in archaea and eukaryotes.

## Results

### Snu13 binds to none of the predicted non-canonical box C’/D’ motifs

Of the 43 known snoRNAs in yeast, only two have box C’/D’ motifs matching the canonical sequences^26^. To understand whether predicted, “non-canonical” box C’/D’ sequences adopt the typical secondary structure and k-turn three-dimensional structure that justifies their annotation as box C’/D’ motifs, we selected three such motifs from the *Saccharomyces cerevisiae* (*Sc*) snoRNAs, snR41, snR51 and snR54, and tested their ability to bind the L7Ae protein of archaea and the Snu13 protein of eukaryotes, which specifically recognize k-turn structures. We annotated the box C’ and D’ sequences of these three snoRNAs manually, as described in Methods. The box D’ sequences corresponded to those annotated in the yeast snoRNA database UMass-Amherst^41^. The box C’ sequences of snR51 and snR54 corresponded to those annotated in^26^. The box C’ sequence annotated for snR41 (UACAUGU**)**, differed by three nucleotides from that annotated in^26^ (AUGUGCA), because our choice maximized the number of base-pairs in stem II matching those of a canonical box C’/D’ motif. We used the sequences from snR51, snR41 and snR54 predicted to form box C’/D’ motifs and their flanking structural elements to generate three RNAs: snR51-kl1, snR41-kl1, and snR54-kl1 (Fig. 1B,C). The RNAs were designed to reproduce the native local structure around the predicted box C’/D’ motifs; we avoided adding sequences that would cause the 3’ and 5’ ends of the RNAs to form a helical structure, as the sequences downstream of box C’ and upstream of box D’ in the snoRNAs do not base-pair with each other. Secondary structure predictions for box C’/D’ elements shown in (Fig. 1B-C) are based on sequence annotations of box C’ and D’, as described above, and experimentally-known, protein-bound secondary structures of box C/D elements^22,42^. Rather than study the structures of these RNAs in isolation, we tested whether they adopt the typical box C’/D’ kinked structure by assaying their ability to bind L7Ae. This protein has a very strong affinity for k-turn-like RNA structures^40^ and also binds box C/D sequences that deviate from the consensus^43,44^, as well as RNAs that do not have a stable fold in isolation (such as sR26-kl, which is derived from the box C’/D’ motif of *P. furiosus* sR26, Fig. 1C). Moreover, we know that even box C/D consensus sequences do not necessarily adopt the k-turn conformation in the absence of binding proteins^45,46^. Also, we tested whether snR51-kl1, snR41-kl1, and snR54-kl1 bound to Snu13, the yeast orthologue of L7Ae. Protein binding to the RNAs was monitored by electrophoretic mobility shift assays (EMSA)^47^ using 5’-Cy5 fluorescently labeled RNAs.

Of the three putative box C’/D’ motifs tested, only snR51-kl1 bound to L7Ae (Fig. 1D). Similar to the positive control sR26-kl, the snR51-kl1 RNA was completely displaced at a 1:1 ratio of the total concentrations of RNA and protein. This demonstrates that the K_D_ of the complex is at least one order of magnitude smaller than the total RNA concentration, corresponding to a K_D_ ≤ 100–200 nM). According to its annotation, the snR51-kl1 box C’/D’ motif contains the 1n−1b A•G base-pair of the consensus sequence, as well as base-pairs –1 and –2 of stem I. By contrast, the predicted snR41-kl1 box C’/D’ motif lacks the 1n−1b A•G base-pair, and snR54-kl1 lacks both base-pair –1 of stem I and the 2n−2b G•A base-pair of stem II. The positive control sR26-kl has the consensus box C/D sequence but lacks stem I.

The results of Fig. 1D suggest that either a conserved box C/D consensus sequence or an intact −1 base-pair in stem I and a conserved 1n−1b A•G base-pair are necessary for the RNA to bind L7Ae. To verify this conclusion, we generated mutants of all three snR51-kl1, snR41-kl1 and snR54-kl1 RNAs and tested their ability to bind L7Ae (Fig. 1D). As predicted, L7Ae interacted with snR41-kl2, which has the 1n−1b A•G and the 2n−2b G•A base-pairs, as well as an intact –1 base-pair in stem I, albeit weaker than with snR51-kl1. Introduction of the 2n−2b G•A base-pair in snR51-kl2 did not improve its affinity for L7Ae, whereas substitution of stem I with a loop in snR51-kl3 increased the dissociation rate (k_off_) of the complex, leading to smearing of the gel bands. On the other hand, snR51-kl4, with the 2n−2b G•A base-pair but without the −1 base-pair, bound to L7Ae similarly to snR51-kl1.

Unlike L7Ae, Snu13 bound none of the three native RNAs, snR51-kl1, snR41-kl1 and snR54-kl1 at the maximum ratio of the total concentrations of RNA and protein of 1:2, which suggested a K_D_ at least one order of magnitude higher than the total RNA concentration (corresponding to a K_D_ ≥  ~ 10 μM). Weak binding with band smearing was seen for the snR51-kl2 mutant containing the 2n−2b G•A base-pair (Fig. 1E).

Finally, we repeated a subset of EMSAs using a 50 times higher RNA concentration of non-fluorescently labelled RNA (Supplementary Fig. 3) and visualized the RNA by staining with ethidium bromide. Also in this case, snR51-kl2 was the only RNA that showed binding to Snu13, confirming that all other RNAs either do not bind Snu13 (K_D_ ≥  ~ 500 μM) or do so with a very weak affinity (K_D_ ~ 100 μM). As a control Snu13 bound canonical k-turn sequences at a ratio of the total concentrations of RNA and protein of 1:1 (Supplementary Fig. 4).

These data suggest that both 1n−1b A•G and 2n−2b G•A base-pairs and an intact −1 base-pair in stem I are required to form a k-turn structure that can be recognized by Snu13. Thus, box C’/D’ sequences containing all three features form a functional box C’/D’ motif in eukaryotes.

### Structure of archaeal L7Ae bound to a eukaryotic non-canonical box C’/D’ motif

**Figure 2:**
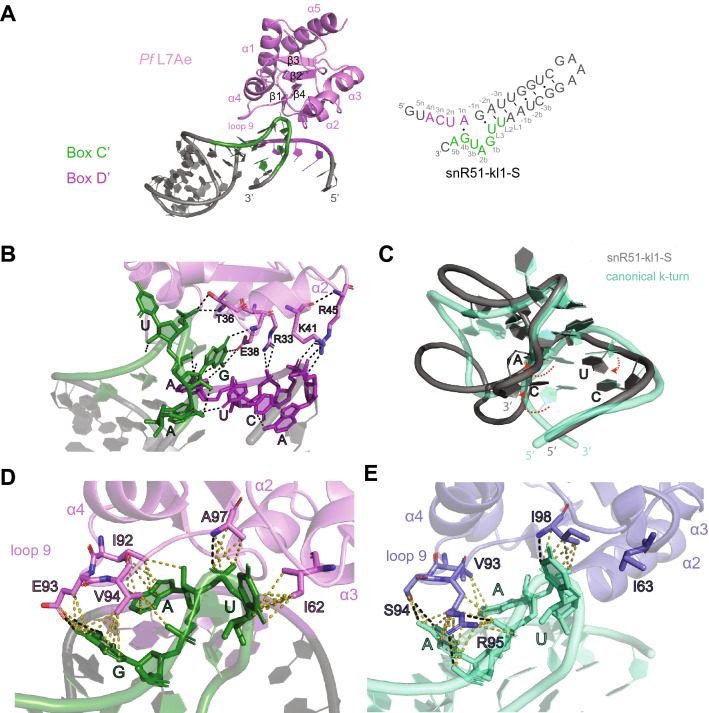
Structure of archaeal L7Ae bound to a eukaryotic non-canonical box C’/D’ motif. (**A**) X-ray structure of *Pf* L7Ae (pink) bound to RNA snR51-kl1-S (gray), containing the predicted box C’/D’ motif from *Sc* guide RNA snR51. Predicted box C’ and D’ are colored in green and purple, respectively. The secondary structure of snR51-kl1-S seen in the X-ray structure deviates from the one predicted in Fig. 1B,C and is shown on the right. (**B**) Interaction of the unpaired box D’ (purple) and C’ (green) nucleotides with L7Ae. Polar contacts and hydrogen bonds are represented as black dashed lines. (**C**) Overlay of the kinked structure formed by the non-canonical box C’/D’ motif of snR51-kl1-S (gray) and the canonical k-turn structure formed by a box C/D motif matching the consensus sequence (mint) (PDB code: 1RLG). Differences in the position of the bases and the RNA backbone are marked by red dashed arrows. (**D**,**E**) Comparison of the interactions of L7Ae (**D**) and Snu13 (**E**) loop 9 amino acids with the snR51-kl1-S RNA (D) and the U4 k-turn RNA (**E**) (PDB code: 1E7K), respectively. Polar interactions and hydrogen bonds are shown as black dashed lines, hydrophobic contacts as yellow dashed lines. In (**D**) box C’ and D’ are colored green and purple, respectively. Structures are displayed with the PyMOL Molecular Graphics System (version 2.0, Schrödinger, LLC; https://pymol.org/2/). The figure was prepared with Adobe Illustrator (Adobe Inc., 2020/21; https://adobe.com/products/illustrator).

The 2n−2b G•A base-pair of stem II, whose absence abolishes binding to Snu13 but not to L7Ae, is conserved in archaeal guide RNA. Thus, to understand whether non-canonical box C’/D’ motifs lacking the 2n−2b G•A base-pair adopt a k-turn-like structure in the presence of L7Ae, we set out to solve the structure of the L7Ae–snR51-kl1 complex. We obtained crystals for L7Ae in complex with a snR51-kl1 mutant, termed snR51-kl1-S, in which stem I was shortened to eight base-pairs and determined the X-ray crystallographic structure at a resolution of 1.9 Å (Fig. 2A, Table 1, Supplementary Fig. 5). The annotated box C’/D’ sequences did not base-pair as predicted. Instead, the 1n adenosine and the 4b guanosine formed the first A•G base-pair of stem II, rather than the 1n and 1b nucleotides. This shifted the putative box C’ sequence by three nucleotides from 5’-UUGAUGA to 5’-AUGACUA (Table S2). Nucleotides originally annotated as L2 and L3 (Fig. 1) were part of stem I, whereas nucleotide 3b was bulged out and adopted the position of L3 in the structures of L7Ae–k-turn RNA complexes^42^. The new base-pair pattern resulted in stem I containing more canonical base-pairs than predicted as well as in a purine, instead of a pyrimidine, at position L2 (Fig. 2A). The preference for a purine in this position had been previously established for the Snu13–U3 box C’/D complex, because of its favorable stacking on the first A•G base-pair of stem II^48^. Finally, as in many crystal structures of protein-RNA complexes or of isolated RNA, we cannot exclude that crystal packing contacts between RNA molecules influence the conformation adopted by the RNA in the crystal (Supplementary Fig. 5). In our structure the stretch ^1^GUAC^4^ of one RNA forms base-pairs with the same stretch of the neighboring RNA molecule.

**Table 1:** Crystallographic data collection and refinement statistics.

Structure	L7Ae―snR51-kl1-S
PDB-ID:	7OZQ
**Data collection**	
Beamline	P11, PETRA III, DESY
Wavelength (Å)	1.03
Space group	C2
**Cell dimensions**	
*a*, *b*, *c* (Å)	109.39, 61.60, 138.61
α, β, γ (°)	90.00, 108,36, 90.00
Resolution (Å)^a^	54.50–1.91 (1.94–1.91)
*R*_merge_ (%)^a,1^	8.1 (77.9)
*R*_pim_ (%)^a,2^	3.4 (32.0)
*I/*σ*I*^*a*^	12.4 (2.1)
Completeness (%)^a^	100 (100)
Redundancy^a^	6.8 (6.8)
CC_1/2_ (%)^a^ (Karplus and Diederichs, 2012)	99.8 (84.9)
**Refinement**	
Resolution (Å)	54.30–1.91
No. reflections	68,473
*R*_work/_*R*_free_ (%)	18.79/22.84
No. atoms	7092
Protein/RNA	6517
Ligand/ion	10
Water	565
B-factors (Å^2^)	41.52
Protein	38.59
RNA	45.19
Ligand/ion	58.65
Water	43.36
**R.m.s deviations**	
Bond lengths (Å)	0.004
Bond angles (º)	0.73
**Ramachandran statistics (%)**	
Favored	99.59
Allowed	0.41
Outliers	0

^a^Values for the highest resolution shell are shown in parentheses.

^1^R_merge_ = Σ_h_ Σ_i_ |< I_h_ >—I_h,i_|/Σ_h_ Σ_i_ I_h,i_, where h enumerates the unique reflections and i are their symmetry-equivalent contributions.

^2^*R*_*pim*_ = Σ_h_ [1/(/n_h_ − 1)]^1/2^ Σ_i_|< I_h_ >—I_h,I_|Σ_h_Σ_i_I_h,I_, where h enumerates the unique reflections and i are their symmetry-equivalent contributions.

In our structure, stem II was disrupted with the exception of the first sheared G•A base-pair. The guanosine of this base-pair engaged the backbone H_N_ and the side chain of L7Ae residue E38 in two crucial hydrogen bonds with its O6 and imino hydrogen, respectively (Fig. 2B). These interactions are conserved in all structures of L7Ae and Snu13 in complex with k-turn RNAs^22,44,49^, explaining why this A•G base-pair is essential for protein recognition. Next to the A•G base-pair was a hydrogen bond formed between the O2 of the U(2n) and the N6 of the A originally annotated as 5b stacked on top of A(1n) (Fig. 2B). There were numerous polar interactions between the box D’ sequence and the Arg and Lys residues of L7Ae helix $\mathrm{\alpha }$2 (Fig. 2B); thus, despite remaining single-stranded, the backbone of the box D’ element was oriented similarly to that of a canonical k-turn, while the backbone of the box C’ element followed a different trajectory (Fig. 2C).

In the crystal structure, the side of stem I in the RNA snR51-kl1-S contacted loop 9 of L7Ae (Fig. 2D). The guanosine residue initially annotated as 1b stacked on the first G•U base-pair of stem I; its imino hydrogen was involved in a hydrogen bond with the side chain of E93, as in all other L7Ae–k-turn RNA complexes with a guanosine residue at this position. Hydrophobic contacts occurred between residues I92 and V94 and the bases of the purines initially annotated as 1b and 2b, respectively. Residues A97 of loop 9 and I62 of helix $\mathrm{\alpha }$4 formed a cluster of hydrophobic residues around the base and backbone of the central kink nucleotide (the 3b uridine), while the backbone carbonyl of D58 formed a hydrogen bond with its imino hydrogen.

We conclude that L7Ae induces a kinked-structure in the RNA even in the absence of the 2n−2b G•A base-pair and when stem II is disrupted. The eukaryotic orthologue Snu13 is unable to do the same and requires the 2n−2b G•A base-pair to bind the RNA. However, even in the presence of L7Ae, the box C’/D’ motif of snR51 is not a *bona fide* box C’/D’ motif, as the backbone of the box C’ sequence adopts a different conformation from that of a k-turn (Fig. 2C).

### Amino acid residues in loop 9 of L7Ae and Snu13 tune binding affinities for guide RNA

The much stricter sequence requirements needed for Snu13 binding, together with the poor conservation of box C’/D’ elements in yeast, suggests that Snu13 has evolved to recognize mostly canonical box C/D motifs.

**Figure 3:**
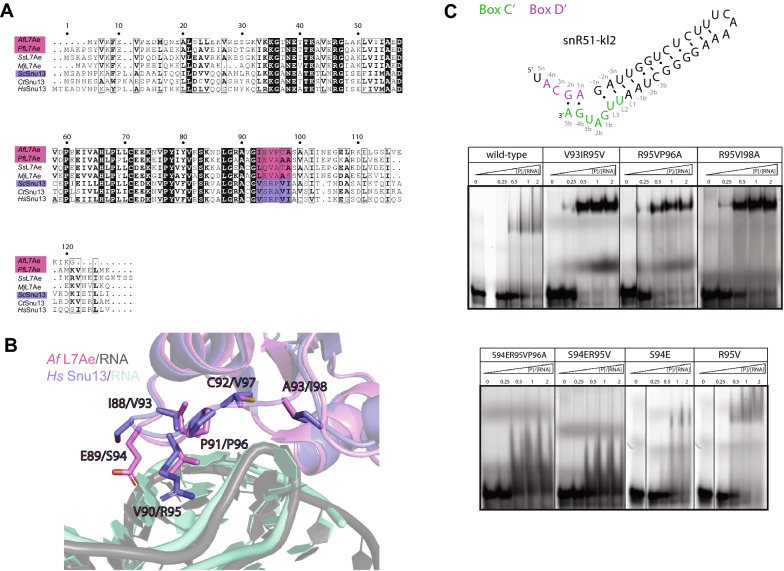
Influence of Snu13 loop 9 on binding affinities for guide RNA. (**A**) Alignment of the primary sequences of archaeal and eukaryotic L7Ae and Snu13 ortholog proteins, showing the poor conservation of loop 9 residues (in pink and purple) from archaea (pink) to eukaryotes (purple). The residue numbering is that of Snu13 from *S. cerevisiae*. *Af – Archaeoglobus fulgidus, Pf* – *P. furiosus*, *Ss* – *S. solfataricus*, *Mj* – *M. jannaschi, Sc – S. cerevisiae Ct* – *C. thermophilum*, *Hs* – *H. sapiens*. (**B**) Overlay of the structure of *Af* L7Ae (pink) in complex with a canonical k-turn RNA (gray) (PDB code: 1RLG) and the structure of *Hs* Snu13 (slate) in complex with the U4 k-turn RNA (turquoise) (PDB code: 1E7K). (**C**) Electrophoretic mobility shift assays of 5’-Cy5 fluorescently labeled snR51-kl2 RNA in the presence of *Sc* Snu13. The predicted secondary structure of the snR51-kl2 RNA is shown at the top. The Snu13 mutants used in the assays are given above each panel. The assays for mutants V93IR95V, R95VP96A, R95V and S94E were run in the same gel and thus share the same control lane; this lane is copied next to the lanes of each individual mutant and separated by a thin line. The first lane of each panel contains RNA only. The ratio of total protein and total RNA concentrations ([P]:[RNA]) is given above each lane. The RNA concentration in each of the five lanes is in the order: 2, 1.9, 1.8, 1.7 and 1.4 µM. The sequence alignment was done with ESPript 3.0.( https://espript.ibcp.fr). Structures are displayed with the PyMOL Molecular Graphics System, (version 2.0, Schrödinger, LLC; https://pymol.org/2/). The figure was prepared with Adobe Illustrator (Adobe Inc., 2020/21; https://adobe.com/products/illustrator).

One difference between Snu13 and L7Ae is the inability of the eukaryotic protein to bind k-loop RNAs, namely box C’/D’ motifs lacking stem I. This difference has been attributed to residues in the protein loop 9^43^, whose sequence diverges significantly between archaea and eukaryotes but is well conserved within each kingdom of life (Fig. 3A). In structures of both L7Ae and Snu13 in complex with canonical k-turn RNAs, loop 9 contacts the major groove of stem I (Fig. 3B). To understand why L7Ae binds to k-loop RNAs while Snu13 does not, we generated seven Snu13 mutants with eukaryotic-to-archaea mutations in loop 9 (S94E, R95V, V93IR95V, S94ER95V, R95VP96A, R95VI98A, and S94ER95VP96A, Fig. 3A,B) and tested their ability to bind the RNAs of Fig. 1C. Three of these mutants had been tested previously for the mouse analogue of Snu13, the protein SNU13 (or 15.5 K), together with the archaeal box C/D and box C’/D’ motifs of sR8 from *Methanococcus jannaschii*^43^*.*

Binding of Snu13 to snR54-kl1 and snR54-kl2 was promoted by the V93IR95V and R95VP96A mutations, while the V93IR95V mutant bound also to snR51-kl3 and snR54-kl3 with a loop instead of stem I (Supplementary Fig. 6). In general, binding to RNAs without a stable stem I was promoted by an increase in the hydrophobicity of loop 9. By contrast, introducing a negative charge, as in Snu13 S94E, lowered the binding affinity (Fig. 3C and Supplementary Fig. 6).

Some of the mutations modulated the affinity of Snu13 for snR51-kl2 (Fig. 3C). The V93IR95V, R95VP96A and R95VI98A mutant bound snR51-kl2 better than wild-type: the appearance of well-defined bands for the complex species in the EMSAs suggested that the increased affinity was due to a decrease in the dissociation rate, k_off_. These results can be rationalized comparing our crystal structure of the L7Ae–snR51-kl1-S RNA complex with the published structure of human Snu13 bound to the U4 RNA k-turn element (PDB ID: 1E7K) (Fig. 2D,E). In complex with the snR51-kl1-S RNA, substitution of L7Ae-V94 by Snu13-R95 would weaken the hydrophobic contacts with the guanine 1b and at the same time compensate this loss with electrostatic contacts to the RNA backbone. Consistent with this, the Snu13 mutant R95V bound to snR51-kl2 with similar affinity as wild type Snu13 did (Fig. 3C). By contrast, the hydrophobic contacts between L7Ae-I92 and the adenine 2b would be weakened if the Ile were substituted by Snu13-V93, explaining why the Snu13 mutant V93IR95V bound snR51-kl2 better than the wild type. The longer side-chain of Snu13-I98, substituting L7Ae-A97, would form more hydrophobic contacts to the bulged-out nucleotide, but would push Snu13-I63 in helix $\mathrm{\alpha }$4 away from the RNA, explaining why the Snu13 mutant R95VI98A binds snR51-kl2 better than wild-type.

Altogether, our experiments confirm the role of loop 9 residues in determining the affinity of the protein for k-loop RNAs but also reveal how the nature of these residues fine-tunes binding affinities to k-turn RNAs.

### Assembly in Box C/D complexes does not rescue non-functional box C’/D’ motifs

**Figure 4:**
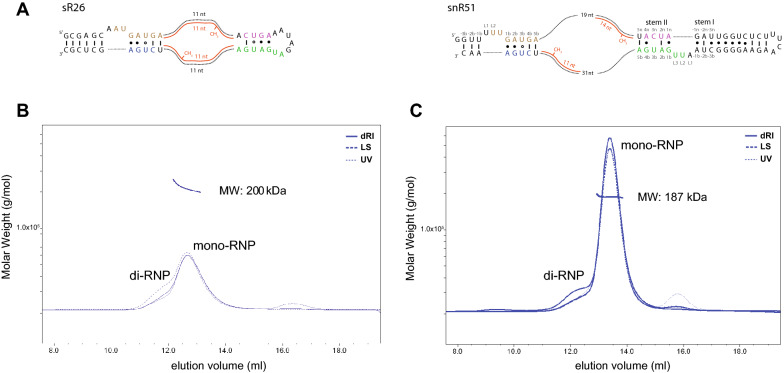
Snu13 does not bind to the putative box C’/D’ motifs of eukaryotic snoRNAs even when they are assembled in Box C/D complexes. (**A**) Sequence and predicted secondary structure of archaeal box C/D guide RNA sR26 and eukaryotic box C/D guide RNA snR51. Box C, box D, box C’ and box D’ are colored in brown, blue, green and purple, respectively. The guide sequences are shown base-paired with the rRNA substrates (red); the length of each complementary guide sequence is given below the substrate RNAs. (**B**) Multi-angle light scattering profile of the RNP containing snR51, Snu13, Nop5 and fibrillarin. (**C**) Multi-angle light scattering profile of the RNP containing sR26, Snu13, Nop5 and fibrillarin. The figure was prepared with Adobe Illustrator (Adobe Inc., 2020/21; https://adobe.com/products/illustrator).

After testing the affinity of isolated Snu13 for non-canonical box C’/D’ sequences, we asked whether this affinity could be modulated by the presence of the scaffolding proteins Nop56/Nop58. Because the heterocomplex of Nop56 and Nop58 cannot be reconstituted from overexpressed proteins in a homogeneous form and sizeable quantities, we sought to answer this question using archaeal Nop5, instead. We reconstituted a chimeric RNP complex containing yeast guide RNA snR51 (Fig. 4A), Snu13 and the complex Nop5_2_–Fib_2_ from the archaea species *P. furiosus*. As a control, we also used the guide RNA sR26 from *P. furiosus*. The in vitro reconstituted complexes were purified by size-exclusion chromatography (Fig. 4B). snR51 formed RNP particles containing Snu13 and Nop5_2_–Fib_2_, demonstrating that Nop5_2_ can substitute for the eukaryotic proteins Nop58 and Nop56 in binding to the Snu13–RNA complex.

We then analyzed the particles assembled with snR51 and sR26 by size-exclusion chromatography and multiple angle light scattering (MALS). The RNP assembled with snR51 (Fig. 4C) resulted in a main peak with a molecular weight (MW) of ~ 187 kDa. This MW corresponds to a monomeric RNP (Supplementary Fig. 2) containing one copy of the Nop5_2_–Fib_2_ tetramer, one copy of the guide RNA and only one copy of Snu13 (theoretical MW 193.7 kDa). A second peak related to a dimeric RNP (di-RNP, Supplementary Fig. 2). This result demonstrates that the presence of Nop5_2_–Fib_2_ does not promote binding of a second copy of Snu13 to the non-canonical box C’/D’ motif of snR51.

A similar elution profile was obtained for the complex assembled with the archaeal sR26 RNA, Nop5_2_–Fib_2_ and Snu13 (Fig. 4B), with the difference that the monomeric and dimeric RNP peaks were partially overlapped, compromising the accurate determination of their molecular weights. Nevertheless, the MW measured by MALS for the right-most part of peak corresponding to the monomeric RNP, is ~ 200 kDa, which fits with a particle containing one copy of the Nop5_2_–Fib_2_ tetramer, one copy of the guide RNA and two copies of Snu13 (theoretical MW 193.3 kDa) rather than only one copy of Snu13 (theoretical MW 179.7 kDa). This data suggests that the presence of Nop5_2_–Fib_2_ promotes binding of Snu13 to canonical k-loop structures, as that formed by sR26.

These findings may explain conflicting literature data on the ability of Snu13 to bind snoRNA box C’/D’ motifs: on the one hand, Snu13 associates with the box C’/D’ motif of human U24 because in this RNA the box C’/D’ motif forms a canonical k-loop^29^; on the other hand, it is unable to bind the non-canonical box C’/D’ motif of *Xenopus* U25 snoRNA^28^.

## Discussion

The archaeal Box C/D RNPs are often used as a proxy for eukaryotic Box C/D RNPs, but the similarities and differences between them remain unclear. One major question is whether the predicted, internal, non-canonical box C’/D’ motifs in eukaryotic snoRNAs bind the box C/D motif-binding protein Snu13, leading to a similar architecture of the box C/D and box C’/D’ protein-assembly sites. In this study, we revisited the question of the existence of box C’/D’ motifs in yeast snoRNAs by defining a functional box C’/D’ motif as two juxtaposed sequences capable of base-pairing (as in Fig. 1A) and of forming, at least when in complex with proteins, the three-dimensional k-turn-like structure typical of these secondary structure elements. We show that functional box C’/D’ motifs exist in some eukaryotic snoRNAs but not in others. When they exist, they recruit a second copy of Snu13 to the RNA; when they do not exist, no second copy of Snu13 is recruited to the complex. Moreover, we determine the features that define a functional box C'/D' motif in eukaryotes and we show that most of the predicted non-canonical box C’/D’ motifs are not functional.

Among the 43 yeast snoRNAs, only two (snR60 and snR70) contain box C’/D’ motifs that match the consensus sequence; six others (snR58, snR65, snR66, snR69, snR71 and U24) contain both the A•G and G•A sheared base-pairs of stem II and the first base-pair of stem I^26^. Eighteen predicted box C’/D’ motifs contain the tandem sheared base-pairs but lack at least the first base-pair of stem I (for example, snR50, snR52, snR63 and snR74) and the remaining 17 lack one of the two sheared base-pairs (for example snR51, snR41, snR54, snR13 and snR38). According to our systematic mutational analysis, Snu13 binding to the box C’/D’ motif requires the presence of both an intact stem I and the tandem A•G sheared base-pairs. Thus, we predict that only snR60 and snR70, and possibly snR58, snR65, snR66, snR69, snR71 and U24, recruit Snu13 to their putative box C’/D’ motif. In humans none of the 32 snoRNAs homologous to yeast sRNAs contains a canonical box C’/D’ and stem I seems to be absent in most cases. Thus, functional box C’/D’ motifs are by no means universal in eukaryotic snoRNAs^50^.

In archaea, the Nop5 dimer directs the methyltransferase, fibrillarin, to the methylation sites by anchoring its C-terminal domains to the box C/D and C’/D’ motifs of the guide RNA, thereby orienting the RNA guide sequences along its coiled-coil domains (Figure S2). The activity and specificity of archaeal 2’-*O*-methylation complexes depend on this bipartite, symmetrical architecture. We show here that a similar bipartite architecture is unlikely to exist in the 2’-*O*-methylation complexes of eukaryotes, which have a number of asymmetric features. It follows that archaeal Box C/D RNPs are not a satisfactory proxy for eukaryotic Box C/D RNPs in all their functional and regulation aspects.

The prediction that no more than 8 of the 43 yeast methylation guide RNAs contain a functional box C’/D’ motif calls into question their role in binding Nop56 and indicates that a RNA three-dimensional structure different from both the k-turn and the k-loop might recognize this protein. Based on the observation that box C’/D’ motifs are not universal in eukaryotic snoRNAs, we propose that the interaction of Nop56 with the guide RNA does not depend on a box C’/D’ motif but on a yet-unknown RNA motif or structure, which has evolved exclusively in eukaryotes from the box D’ sequence and may partially overlap with it. In this scenario, the box C/D sequence would have a leading role in initiating complex assembly by the recruitment of Snu13 and Nop58, followed by a chaperone-aided dimerization of Nop58 with Nop56. Only at this point Nop56, readily recruited to the complex, would be able to recognize the RNA next to the box D’ site in a yet-unknown manner. It is also possible that the C-terminal domain of Nop56 is not involved in RNA binding and that the guide-substrate RNA duplex is recognized in the right register by the Nop56-N-terminal-domain-fibrillarin complex with the help of transiently-associated chaperones.

The role of the box C/D element in initiating the assembly of snoRNPs is supported by functional data in vivo. In a study of the effect of mutation or depletion of various secondary structure elements in an engineered guide snoRNA on rRNA methylation in yeast^29^, mutation or depletion of the box C/D motif abolished methylation at both sites upstream of box D and box D’. By contrast, depletion of box D’ (or box C’) affected methylation only upstream of box D’^29^, indicating that the box C’/D’ motif is required neither for the assembly of a functional complex nor for methylation specificity at the site upstream of box D. The sequence of the engineered snoRNA used in this study was derived from human U24, which has a canonical box C’/D’ k-loop sequence and was shown to recruit two copies of Snu13. The fact that even in this case the conserved box C’/D’ element is unable to nucleate the assembly of the methylation complex indicates that in vivo the snoRNP does not adopt a symmetric bipartite structure like the archaea Box C/D RNPs. These findings and our own data together strongly suggest that the architecture of Box C/D snoRNPs at the box C/D and (putative) box C’/D’ sites differ from each other. This conclusion is not in the disagreement with the symmetric architecture of the U3 snoRNP bound to the pre-ribosomal 90S subunit^36^, as the U3 snoRNP does not function as a methyltransferase and the U3 RNA has two canonical box C/D sequences, unlike most of the methylation competent snoRNAs. The apparent existence of different assembly modes for Snu13, Nop56 and Nop58 on Box C/D snoRNAs demonstrates the versatility of the eukaryotic snoRNP machinery: the sequence of the snoRNA determines the assembly mode at the Nop56 site to support different functions.

The idea that the symmetric bipartite architecture seen in archaeal Box C/D RNPs does not exist in methylation-competent eukaryotic snoRNPs is further supported by the biochemical and in vivo evidence of the importance of the spacer/guide sequences between box C and box D’ or box C’ and box D in archaea and eukaryotes. In archaea, the optimal spacer/guide length is 12 nucleotides; alteration of the length of one of the two spacer/guides impacts the methylation only of the corresponding substrate, in agreement with similar architectures at the box C/D and box C’/D’ sites^51^. In eukaryotes, the picture is less clear cut: methylation of the substrate upstream of box D is (moderately) sensitive to alterations of the spacer/guide length between box C’ and box D, whereas methylation of the substrate upstream of box D’ is sensitive to alterations of both spacer/guide sequence lengths^29^.

In summary, we show that a functional box C’/D’ element does not exist in most yeast guide RNAs, leading us to conclude that this RNA element is not the specific recognition motif for Nop56. We propose that eukaryotic, methylation-competent RNPs have a non-symmetric architecture with different protein–RNA contacts and assembly geometries at the box C/D and putative box C’/D’ sites.

The asymmetric nature of the eukaryotic complex may result into different mechanisms for the regulation of methylation levels at the substrates upstream of box D and box D’ and thus into a higher flexibility for the coupling of site-specific methylation to other cellular processes.

## Material and methods

### Cloning and mutagenesis

Genes encoding full-length L7Ae, Nop5, and fibrillarin in *P. furiosus* (UniProtKB accession code Q8U160, Q8U4M1, and Q8U4M2) were obtained by PCR from genomic *P. furiosus* (*Pf*) DNA. The genes were cloned into expression vector pET-M11 containing a TEV (tobacco etch virus) protease-cleavable N-terminal His_6_-Tag using BamHI and NcoI restriction sites^31^. The full-length *SNU13* gene from *S. cerevisiae* (UniProtKB accession code P39990) was ordered from Invitrogen with codon-usage optimized for *E. coli* translation. The gene was amplified via PCR and cleaved with NcoI and XhoI restriction enzymes (New England Biolabs, NEB). Cleaved PCR products were ligated into the cleaved pET-M11 expression vector. The final Snu13 construct contained an N-terminal His_6_-Tag cleavable with TEV-protease.

Snu13 point mutations were accomplished using the Pfu Plus! DNA Polymerase (Roboklon) according to the protocol provided by the manufacturer. PCR products were cleared from the starting material by DpnI (NEB) digest; the enzyme was heat-inactivated before transformation of the cleared PCR products into *E. coli* OmniMax cells. Positive mutants were verified by sequencing (Eurofins).

Full-length DNA templates for *S. cerevisiae* (*Sc)* guide RNAs snR51 (Gene-ID: 9.164.983), snR41 (Gene-ID: 9.164.986), and snR54 (Gene-ID: 9.164.960) were ordered as synthetic genes in cloning vector pUC57 from GENEWIZ (Sigma-Aldrich). All templates contained a 3’ PstI cleavage site for DNA linearization. For all other RNA constructs, the template DNA was ordered as single-stranded DNA with EcoRI (5’ GAATTC) and HindIII (5’ AAGCTT) cleavage sites at the 5’- and 3’-end, respectively, as well as a PstI cleavage site upstream of the HindII site. Complementary single-stranded DNA molecules were annealed, cleaved with EcoRI-HF, and HindII-HF (NEB), and purified with the QIAquick PCR purification kit (Qiagen). The inserts were ligated into a cleaved pUC19 cloning vector using T4 DNA ligase (NEB). Correct insertion was verified by sequencing (Eurofins).

### Protein expression and purification

His_6_-tagged L7Ae, Nop5, and fibrillarin (Fib) were expressed in *E. coli* BL21(DE3). Cells were grown in LB Medium at 37 °C until an OD_600_ of 0.6–0.8, and expression was induced at 20 °C with 1 mM final concentration of IPTG (Carl Roth). Cells were harvested 18–20 h after induction by centrifugation at 4500 rpm and 4 °C.

The cell pellet was resuspended in lysis buffer A (50 mM Tris–HCl, 1 M NaCl, 10% glycerol, 10 mM imidazole, 10 mM $\upbeta$-mercaptoethanol, pH 7.5) complemented with one tablet of cOmplete, EDTA-free protease inhibitor cocktail (Roche). After addition of 1 mg lysozyme (Carl Roth), the resuspended cell pellet was incubated for 30 min on ice; afterwards, the cells were lysed by 30 min sonication on ice. The lysate was cleared by centrifugation at 18,500 rpm and 16 °C for 1 h. For L7Ae, the supernatant was mixed with lysis buffer containing 8 M guanidinium hydrochloride (GdnHCl) in a 1:3 ratio to a final GdnHCl concentration of 6 M. The denatured lysate was loaded on a 5 ml HisTrap FF column (Cytiva) using an Äkta Pure system with an external sample pump. After sample loading, the bound protein was refolded by reducing the GdnHCl concentration stepwise with 20 column volumes of lysis buffer. The refolded L7Ae was eluted in 0 M GdnHCl and up to 50% buffer B (50 mM Tris–HCl, 1 M NaCl, 10% glycerol, 1 M imidazole, 10 mM $\upbeta$-mercaptoethanol, pH 7.5). For Nop5 and fibrillarin, the supernatant was boiled for 15 min at 80 °C and cleared by centrifugation at 18,500 rpm and 16 °C. The supernatant was loaded onto a 5 ml HisTrap FF column, which was washed six times with 3 column volumes of high-salt buffer C (50 mM Tris–HCl, 1 M NaCl, 10% glycerol, 10 mM imidazole, 2 M LiCl, 10 mM $\upbeta$-mercaptoethanol, pH 7.5). The proteins were eluted with buffer B. After elution, all proteins (L7Ae, Nop5, and fibrillarin) were buffer-exchanged into buffer A using a HiPrep 26/10 desalting column (Cytiva). The N-terminal His_6_-Tag was removed by overnight cleavage with TEV-protease (produced in-house) at room temperature. The reaction mixture was loaded on a 5 ml HisTrap FF column, which retained the TEV-protease and the cleaved His_6_-Tag, while the cleaved protein was collected with the flow-through.

His_6_-tagged Snu13 and Snu13 mutants were expressed in *E. coli* BL21 (DE3). The transformed cells were grown in LB medium at 37 °C until an OD_600_ of 0.6–0.8, and expression was induced at 16 °C with 1 mM final concentration of IPTG. Cells were harvested 18–20 h after induction by centrifugation at 4500 rpm and 4 °C. The cells were then resuspended in lysis buffer D (50 mM Tris–HCl, 1 M NaCl, 5% glycerol, 10 mM imidazole, 10 mM $\upbeta$-mercaptoethanol, pH 7.5) complemented with one tablet of cOmplete, EDTA-free protease inhibitor cocktail. After addition of 1 mg lysozyme (Carl Roth) the resuspended cell pellet was incubated for 30 min on ice and then lysed by 30 min sonication on ice. The lysate was cleared by centrifugation at 18,500 rpm and 16 °C for 1 h. The supernatant was loaded on a 5 ml HisTrap FF column, which was washed six times with three column volumes of high-salt buffer E (50 mM Tris–HCl, 1 M NaCl, 5% glycerol, 10 mM imidazole, 2 M LiCl, 10 mM $\upbeta$-mercaptoethanol, pH 7.5). The protein was eluted using a gradient up to 50% of buffer F (50 mM Tris–HCl, 1 M NaCl, 5% glycerol, 1 M imidazole, 10 mM $\upbeta$-mercaptoethanol, pH 7.5) and subsequently buffer exchanged into buffer G (50 mM Tris–HCl, 150 mM NaCl, 5% glycerol, 10 mM $\upbeta$-mercaptoethanol, pH 8.0) using a HiLoad desalting 26/10 column. To remove bound RNA, the eluate was loaded on a 5 ml QTrap HP column (Cytiva), from which the RNA-free protein was collected with the flow-through. The RNA and RNA-bound protein were eluted from the column with buffer H (50 mM Tris–HCl, 2 M NaCl, 5% glycerol, 10 mM $\upbeta$-mercaptoethanol, pH 8.0). The RNA-free protein was cleaved with TEV protease (produced in-house) to remove the N-terminal His_6_-Tag. The TEV protease and the cleaved tag were removed by affinity chromatography using a 5 ml HisTrap FF column.

The purity of all proteins was confirmed by SDS gel electrophoresis and size exclusion chromatography (SEC).

### snoRNA sequence annotation

Yeast snoRNA sequences were obtained from the yeast snoRNA database UMass-Amherst^41^. All snoRNAs considered here have experimentally verified methylation sites^52^. Box D and D’ motifs were manually annotated using the consensus motif 5’-CUGA and had to start five nucleotides downstream of the canonical base-pair formed between the snoRNA and the target nucleotide in the rRNA. Our annotation was identical to that of the yeast snoRNA database UMass-Amherst^41^. Box C was annotated using the consensus sequence 5’-RUGAUGA and had to start within 5 nucleotides upstream of the 5’ end. Box C’ was annotated manually using the consensus sequence 5’-RUGAUGA and had to be positioned between the box D’ motif and the guide sequence upstream of the box D motif. Our annotation corresponded to that reported in^26^ for all snoRNAs but snR41. For snR41 we chose the sequence 5’-UACAUGU instead of 5’-AUGUGCA as in^26^, because it yielded a stem II that was more similar to that of a genuine box C’/D’ motif than the sequence chosen in^26^.

### RNA transcription

All RNAs used for crystallization and the guide RNAs used for activity assays were produced by in vitro transcription, using T7 RNA polymerase produced in-house. Plasmids containing DNA templates were transformed into *E. coli* Top10; transformed cells were grown in LB medium overnight at 37 °C and harvested by centrifugation at 4500 rpm and 4 °C. Plasmids were extracted using the Qiagen Plasmid Mega Kit (Qiagen) and cleaved with PstI-HF (NEB). Linearized plasmid DNA was purified by phenol/chloroform/isoamyl alcohol and chloroform/isoamyl alcohol (Carl Roth) extraction and concentrated by precipitation with pure ethanol and NaCl.

For the transcription of each RNA construct, the concentrations of DNA, nucleoside triphosphates (NTPs, Carl Roth), MgCl_2_ and T7 polymerase were optimized to maximize the yield. Large-scale transcription reactions were run for five hours at 37 °C. All RNAs were purified using preparative, denaturing polyacrylamide gels containing 8 M urea. Purity was verified using analytical denaturing polyacrylamide gels.

### Electrophoretic mobility shift assays

All RNAs used for the electrophoretic mobility shift assays (EMSA) were purchased from Integrated DNA Technologies (IDT) with a 5’-Cy5 label for fluorescence detection. Three nucleotides were added at the 5’ end as a spacer between the Cy5 label and the desired RNA sequence (Supplementary Table 1). To ensure that the spacer nucleotides did not interfere with the RNA structure, a subset of binding assays were repeated with non-labelled RNAs lacking the spacer nucleotides and analyzed using ethidium bromide. The results were equivalent in all cases.

In the fluorescence-detected binding assays, 10 pmol of RNA were mixed with sterile LC–MS grade water (Merck) and annealing buffer (final concentrations: 10 mM Tris–HCl, 100 mM NaCl, 1 mM EDTA, pH 7.5) in a total volume of 5 µl and annealed by heating to 80 °C and slow cooling to 4 °C in a T100 Thermocycler (Bio-Rad). After annealing, 0, 2.5, 5, 10 and 20 pmol of protein were added and incubated for up to 30 min at 4 °C. Afterwards, 5 × native loading dye (50 mM Tris–HCl, 0.25% xylene cyanol, 0.25% bromophenol blue, 30% glycerol, pH 7.5) was added to each sample. All samples were analyzed on 10% native polyacrylamide gel at 4 °C. Each gel was pre-run for 0.5–1 h before sample loading. After sample loading, gels were run overnight at 4 °C and 10 mA. Gels were analyzed using a Typhoon Trio system (GE Healthcare) with a 670 nm-bandpass (670 BP 30) emission filter for Cy5 detection. Intensities were extracted and analyzed using Fiji^53^.

A subset of EMSAs (L7Ae or Snu13 with sR26-kl, snR51-kl1, snR51-kl2, snR41-kl1, snR41-kl2, snR54-kl1 and snR54-kl2) was repeated with non-labeled RNAs, lacking the spacer nucleotides between the RNA and the dye at the 5' end. In these assays, 0.5 nmol of non-labeled RNA were mixed with pure and sterile LC–MS grade water (Merck) and annealing buffer (final concentrations: 10 mM Tris–HCl, 100 mM NaCl, 1 mM EDTA, pH 7.5) in a total volume of 5 μl and annealed by heating to 80 °C for 1 min and slow cooling to 4 °C in a T100 Thermo Cycler (Bio-Rad). After annealing, 0, 0.125, 0.25, 0.5 and 1 nmol of protein (L7Ae or Snu13) were added, and the mixture was incubated for 30 min at 4 °C. All samples were further were analyzed on 10% native polyacrylamide gel at 4 °C to prevent diffusion and degradation of the protein and the RNA. Gels were stained with ethidium bromide, and the RNA was visualized using a Gel Doc XR + gel documentation system (Bio-Rad).

### Crystallization

Purified archaeal L7Ae and snR51-kl1-S RNA were mixed in a 1:1 ratio, incubated for 15 min at 80 °C and slowly cooled down to room temperature. The protein–RNA complex was purified in crystallization buffer (50 mM MOPS, 50 mM NaCl, 1 mM DTT, pH 6.6) using a HiLoad 16/600 Superdex 75 pg column (Cytiva) on an Äkta Pure system. The purified complex was concentrated using Amicon Ultra-15 3 K centrifugal filters (Merck).

A concentrated solution of ~ 10 mg/ml of L7Ae–snR51-kl1-S was used for crystallization by sitting drop vapor diffusion. Initial crystallization screens were set up with a Crystal Phoenix crystallization robot (Art Robbins Instruments) using NeXtal DWBlock Suites (Qiagen); JCSG Core I Suite, JCSG Core II Suite, JCSG Core II Suite, JCSG Core IV Suite, Nucleix Suite, PEG Suite, and PEG II Suite. The drop solution was equilibrated against 200 µl of reservoir solution at 18 °C. Crystals appeared after one week in several conditions across all initial screens. The best crystal was obtained in 0.02 M CaCl_2_, 0.1 M sodium acetate, 30% 2-Methyl-2,4-pentandiol (MPD) (G12 from Qiagen JCSG Core I Suite). Cryo-protection was achieved by the addition of 10% (2*R*, 3*R*)-2,3-butanediol before flash-freezing.

### Crystallographic data collection and processing

Data were collected at beamline P11 of the PETRA III storage ring, DESY (Deutsches Elektronen-Synchroton, Hamburg, Germany)^54^. The dataset yielding the structure was recorded at 100 K and a wavelength of 1 Å and processed using the AutoPROC toolbox (Global Phasing)^55^ executing XDS^56^, Pointless^57^, Aimless^58^ from the CCP4 program suite^59^. The high-resolution cut off was determined using a signal/noise ratio (I/σ(I)) of 2.0.

### Structure determination

The number of molecules in the asymmetric unit was determined using Xtriage^60^ from the Phenix software package^61^. L7Ae from *P. furiosus* (PDB-ID: 4WB0, sequence identity: 100%) was identified as a suitable search model for molecular replacement by executing Balbes^62^ from the CCP4 program suite^59^. The molecular replacement solution containing only the protein component was improved by executing the AutoBuild tool^63^ from the Phenix software package^61^. The missing RNA component was then built by the AutoBuild tool around the fixed model of the improved protein component. The crystal structure of the protein-RNA complex was further improved and finalized by iterative cycles of model building in Coot^64^ and refinement in Phenix.refine^65^. Data collection and refinement statistics are summarized in Table 1.

### Complex assembly

All RNP complexes were assembled in complex buffer (20 mM sodium phosphate, 500 mM NaCl, pH 6.6) using a Superdex S200 Increase 10/300 GL (Cytiva) on an Äkta Pure system. Nop5 and fibrillarin were mixed in a 1:1.1 ratio, incubated for 15 min at 80 °C and cooled to room temperature^31^. The Nop5_2_–Fib_2_ complex was purified by size-exclusion chromatography. The guide RNA (Supplementary Table 2) was annealed in annealing buffer (10 mM Tris–HCl, 100 mM NaCl, 1 mM EDTA, pH 7.5) at 90 °C for 2 min followed by snap-cooling. Nop5_2_–Fib_2_, Snu13, and the guide RNA were mixed in a 1:2:1 ratio and incubated for 15 min before purification via size-exclusion chromatography.

### Multi-angle-light scattering

Multi-angle-light scattering (MALS) data were collected using an on-line SEC-MALS set-up, consisting of a Superdex S200 Increase 10/300 GL column on an Åkta pure system coupled to a MALS miniDawn TREOS system and Optilab T-rex refractive index detector (Wyatt Technologies). Data were analyzed using the ASTRA 7.0 software package (Wyatt Technologies).

## Supplementary Information

Supplementary Information.

## Data Availability

The structure of the L7Ae–snR51-kl1-S complex is available from the Protein Data Bank under the access code 7OZQ.
