# Supplementary material for: Eukaryotic Box C/D methylation machinery has two non-symmetric protein assembly sites
Source: Sci Rep. 2021 Sep 2;11:17561. doi: 10.1038/s41598-021-97030-y (PMC8413462; doi:10.1038/s41598-021-97030-y)
Supplement: Supplementary file 1 — Supplementary Information. [file 41598_2021_97030_MOESM1_ESM.docx]

**Eukaryotic Box C/D methylation machinery has two non-symmetric protein assembly sites**

Simone Höfler^1^, Peer Lukat^2^, Wulf Blankenfeldt^2,4^, and Teresa Carlomagno^1,3,^*

^1^Institute for Organic Chemistry and Centre of Biomolecular Drug Research (BMWZ), Leibniz University Hannover, Hannover, Lower Saxony, 30167, Germany

^2^ Department of Structure and Function of Proteins, Helmholtz Centre of Infection Research, Braunschweig, Lower Saxony, 38124, Germany

^3^ Group of NMR-based Structural Chemistry, Helmholtz Centre of Infection Research, Braunschweig, Lower Saxony, 38124, Germany

^4^Institute for Biochemistry, Biotechnology and Bioinformatics, Technische Universität Braunschweig, Spielmannstr. 7, 38106 Braunschweig, Germany

**SUPPLEMENTARY MATERIAL**

**SUPPLEMENTARY TABLES**

**Supplementary Table 1 -** Synthetic RNAs used in EMSAs. All RNAs carry a 5’-Cy5 fluorescent label for detection and quantification. The three underlined nucleotides were added at the 5’ end of each RNA as a spacer between the Cy5 label and the desired RNA sequence.

| RNA | Sequence ( 5' 🡪 3') |
| --- | --- |
| sR26-kl | Cy5 - AUAACUGAAAUAGAUGAUGA |
| snR51-kl1 | Cy5 - AUAUACUAGAUUGGUCUCUUUCAAGAAGGGGCUAAUUGAUGA |
| snR51-kl2 | Cy5 - AUAUACGAGAUUGGUCUCUUUCAAGAAGGGGCUAAUUGAUGA |
| snR51-kl3 | Cy5 - AUAUACUAAAUAGUUGAUGA |
| snR51-kl4 | Cy5 - AUAUACGAAAUAGUUGAUGA |
| snR41-kl1 | Cy5 - UAUACUGAAUAGUGGUUUAACUACAUGU |
| snR41-kl2 | Cy5 - UAUACUGAAUAGUGGUUUAACUAGAUGU |
| snR41-kl3 | Cy5 - UAUACUGAAAUAGUACAUGU |
| snR54-kl1 | Cy5 - AUAACUGACUGUGAUCAAACGAUCUUGUAGA |
| snR54-kl2 | Cy5 - AUAACUGACUGUGAUCAAACGAUCUUGAAGA |
| snR54-kl3 | Cy5 - AUAACUGAAAUAGUUGUAGA |

**Supplementary Table 2 -** Sequences of guide RNAs used to assembly chimeric Box C/D RNP complexes. Box C and D are underlined; predicted box C’ and D’ are bold and underlined. The revised box C’ sequence, as seen in the crystal structure of the L7Ae–snR51-kl1-S complex, is indicated in cursive in the sequence of snR51.

| **guide RNA** | **Sequence** |
| --- | --- |
| sR26 | 5' - GCGAGCAAUGAUGAGUGAUGGGCGAA**CUGA**AAUAG**AUGAUGA** CGGAGGUGAUCUCUGAGCUCGC - 3’ |
| snR51 | 5' - GGUUUAUGAUGAUUUUUUUAUAUUCACACUGU**ACUA**GAUUGG UCUCUUUAACGAAGGGGCUAA**UUG*AUGA****CUA*CAAAAUAAAAAAUAACUGAUUUAAUGACUCUGAAAC - 3’ |

**SUPPLEMENTARY FIGURES**

**Supplementary Figure 1 - Structure of canonical box C’/D’ motif bound to L7Ae.** X-ray structure of the archaeal protein L7Ae from *Archaeoglobus fulgidus* bound to an RNA containing a canonical box C/D motif and folding into a k-turn structure (PDB code: 1RLG). Boxes C and D are colored brown and blue, respectively. The sequence and secondary structure of the RNA molecule are shown above the structure.

**Supplementary Figure 2 - Schematic representation of archaeal and eukaryotic Box C/D RNP complexes.** (A) Cartoon models of archaeal Box C/D RNPs. Both the monomeric (mono-RNP) and dimeric (di-RNP) models have been reported, in dependence of the RNA guide sequences. The main components of the complexes are the guide RNA (black), the primary RNA-binding protein L7Ae (wine red), the homodimeric scaffolding protein Nop5 (gray) and the methyltransferase fibrillarin (green). The Nop5 N-terminal domain (NTD) recruits fibrillarin (Fib) to the RNP, while the Nop5 C-terminal domain (CTD) interacts with the composite surface formed by L7Ae and the sRNA. The coiled-coil (CC) domains of two Nop5 monomers dimerize. (B) Models proposed for eukaryotic Box snoRNP complexes. The main components are the guide snoRNA (black), the primary RNA-binding protein Snu13 (blue), the heterodimeric scaffolding proteins Nop56 (turquoise) and Nop58 (green) and the methyltransferase Nop1 (brown). Similar to archaea, the N-terminal domains of Nop56 and Nop58 recruit Nop1 and the C-terminal domains interact either with the Snu13–RNA complex or with the RNA only. In model 1 one copy of Snu13 is recruited to the box C/D motif, while the box C’/D’ motif does not bind Snu13; in model 2 both box C/D and box C’/D’ motifs recruit a copy of Snu13, as seen in the U3 snoRNP in the context of the 90S pre-ribosome.

**Supplementary Figure 3.** Electrophoretic mobility shift assays (EMSAs) of the non-labelled RNAs of Figure 1C in the presence of archaeal L7Ae **(A)** and in the presence of yeast Snu13 **(B)**. The ratio of total protein and total RNA concentrations ([P]/[RNA]) is given above each lane. The RNA concentration in each of the five lanes is in the order: 100, 97, 94, 88 and 78 µM. At these higher concentrations snR51-kl1 and snR51-kl2 form 2:1 protein–RNA complexes with L7Ae and Snu13, respectively.

**Supplementary Figure 4. (A)** Electrophoretic mobility shift assays (EMSAs) of a canonical kink-turn element derived from the snR41 box C/D sequence (5' GGCCCCUAUGAUUAGAAAUAUGAGGGGCC 3') in the presence of yeast Snu13. The ratio of total protein and total RNA concentrations ([P]/[RNA]) is given above each lane. The RNA concentration in each of the three lanes is, in the order: 90, 64, 50 µM. **(B)** Electrophoretic mobility shift assays (EMSAs) of a canonical kink-turn element derived from the snR51 box C/D sequence (5' GGGGUUUAUGAUGAGAAAUCUGAAACCCC 3') in the presence of archaeal L7Ae and yeast Snu13. Each lane contains 0.3 nmol RNA and either 0 or 0.6 nmol protein. The RNA concentration in each of the three lanes is, in the order: 30, 27, 23 µM.

**Supplementary Figure 5. Structure of archaeal L7Ae bound to a eukaryotic non-canonical k-turn structure.** (A) View of the crystal packing of the *Pf* L7Ae protein (violet) and the snR51-kl1-S RNA (green) along the a-axis. (B) View of the crystal packing along the b-axis. (C) View of the crystal packing along the c-axis. (D) Contacts of the snR51-kl1-S RNA (green) bound to *Pf* L7Ae (violet) with symmetry-related RNA molecules (grey). Polar contacts between RNA chains are shown as dashed orange lines. The stretch ^1^GUAC^4^ of one RNA molecule base-pairs with the stretch C^4^–G^1^ of the neighboring RNA molecule. (C) X-ray structure of *Pf* L7Ae bound to the putative Box C’/D’ element of snR51-kl1-S derived from *Sc* guide RNA snR51. L7Ae and RNA are shown in pink and grey, respectively; box C’ and D’ are in green and purple, respectively. The shown 2mF_obs_-DF_calc_ electron density (blue mesh, σ = 1) for the RNA resulted from molecular replacement using the *Pf* L7Ae protein alone.

**Supplementary Figure 6 - Electrophoretic mobility shift assays of Snu13 mutants with native box C’/D’ elements and mutants.** Electrophoretic mobility shift assays performed with Snu13 mutants V93IR95V, R95VP98A, R95VI98A, S94E-R95VP96A, S94ER95V, S94E and R95V and 5’-Cy5 labeled RNA constructs sR26-kl, snR51-kl1, snR51-kl3, snR41-kl1, snR41-kl2, snR41-kl3, snR54-kl1, snR54-kl2 and snR54-kl3. The first lane of each assay contains 10 pmol of RNA only. The ratio of total protein and total RNA concentrations ([P]/[RNA]) is given above each lane. The RNA concentration in each of the five lanes is in the order: 2, 1.9, 1.8, 1.7 and 1.4 µM. When the control lanes were not run next to the titration lanes, the control lane of the same gel is pasted next to the titration lanes and separated by a thin line. The blue asterisk marks RNA degradation products.
